# Supplementary material for: Cultural values and cross-cultural video consumption on YouTube
Source: PLoS One. 2017 May 22;12(5):e0177865. doi: 10.1371/journal.pone.0177865 (PMC5439684; doi:10.1371/journal.pone.0177865)
Supplement: S1 Table — (PDF) [file pone.0177865.s001.pdf]

Country names included and excluded for analyses.

Countries included in the regression			
ARE	United Arab Emirates	KEN	Kenya
ARG	Argentina	KOR	Korea, Republic of
AUS	Australia	KWT	Kuwait
AUT	Austria	LBN	Lebanon
BEL	Belgium	LTU	Lithuania
BGR	Bulgaria	LVA	Latvia
BRA	Brazil	MAR	Morocco
CAN	Canada	MEX	Mexico
CHE	Switzerland	MYS	Malaysia
CHL	Chile	NGA	Nigeria
COL	Colombia	NLD	Netherlands
DEU	Germany	NOR	Norway
DNK	Denmark	NZL	New Zealand
EGY	Egypt	PER	Peru
ESP	Spain	PHL	Philippines
EST	Estonia	POL	Poland
FIN	Finland	PRT	Portugal
FRA	France	RUS	Russian Federation
GBR	United Kingdom	SAU	Saudi Arabia
GHA	Ghana	SEN	Senegal
HKG	Hong Kong	SGP	Singapore
HRV	Croatia	SRB	Serbia
IDN	Indonesia	SVN	Slovenia
IND	India	SWE	Sweden
IRL	Ireland	THA	Thailand
ISR	Israel	TUR	Turkey
ITA	Italy	TWN	Taiwan, Republic of China
JOR	Jordan	USA	United States of America
JPN	Japan	ZAF	South Africa
Countries excluded in the regression			
BHR	Bahrain	OMN	Oman
BIH	Bosnia and Herzegovina	QAT	Qatar
CZE	Czech Republic	ROU	Romania
DZA	Algeria	SVK	Slovakia
GRC	Greece	TUN	Tunisia
HUN	Hungary	UGA	Uganda
MKD	Macedonia, Republic of	UKR	Ukraine
MNE	Montenegro	YEM	Yemen
