# Supplementary material for: Cultural values and cross-cultural video consumption on YouTube
Source: PLoS One. 2017 May 22;12(5):e0177865. doi: 10.1371/journal.pone.0177865 (PMC5439684; doi:10.1371/journal.pone.0177865)
Supplement: S1 Text — (PDF) [file pone.0177865.s002.pdf]

OLS regression analysis using a composite measure of cultural openness.

We measured country C's "cultural openness" using a composite measure that is similar to Rao-Stirling diversity, based on the pairwise overlap in popular videos between C and other countries. Conceptually, cultural openness has three dimensions: (1) *breadth*, indicating the number of countries with which C overlaps on at least one video; (2) *spread*, indicating a uniform distribution in the number of overlapping videos with each of those countries; and (3) *heterogeneity*, which measures the extent to which pairwise embeddedness among countries that overlaps popular videos with C. C's cultural openness is the product of breadth, spread, and heterogeneity, such that a country that consumes the same number of videos (*spread*) in common with many other countries (*breadth*) in culturally different clusters (*heterogeneity*) will have a high openness score, whereas a country that disproportionally consumes videos that are popular in a few countries in culturally similar clusters of countries will have a low diversity score.

Operationally, the openness of country C is the weighted product:

$$\sum_{i,j \in N(C)} w(C,i) \times w(C,j) \times d(i,j)$$

where $N(C)$ denotes *breadth* as the list of countries with at least one popular video co-listed with C. In this formulation, $w(C,i)$ is the normalized fraction of country C's overlapping videos with another country i. The distance $d(i, j)$ indicates Jaccard

pairwise dissimilarity between countries *i* and *j* regarding consumption heterogeneity, the overlap of popular video co-listing countries (i.e., $1 - (N(i) \cap N(j))/(N(i) \cup N(j)))$.

Table 1. OLS regression model of cultural openness among 58 countries.

	**Full model**	**Non-culture Model**	**Culture Model**
**Intercept**	0.317	0.496***	0.411**
	(0.161)	(0.076)	(0.138)
**Non-cultural factors**			
GDP per capita (log-transformed)	-0.117	-0.191	
	(0.206)	(0.237)	
Language eigenvector centrality	0.003	0.171*	
	(0.08)	(0.081)	
Number of Internet users	0.258	0.296	
	(0.225)	(0.252)	
**Cultural values**			
Individualism (IDV)	0.295*		0.356**
	(0.130)		(0.116)
Uncertainty avoidance (UAI)	-0.449***		-0.441***
	(0.114)		(0.106)
Power distance (PDI)	0.450**		0.422**
	(0.146)		(0.138)
Masculinity (MAS)	0.226		0.188
	(0.127)		(0.116)
**Sample size (number of countries)**	58	58	58
**Model-fit indices**			
R^2	0.417	0.103	0.396
Adjusted R^2	0.336	0.053	0.350

Note: * $p < .05$, ** $p < .01$, *** $p < .001$. Unstandardized coefficients are reported with standard errors in parentheses. In order to compare coefficients, variables included in the analyses were rescaled to the unit interval, meaning that the minimum value is 0 and the maximum value is 1.

We tested the model for heteroscedasticity and multicollinearity and found heteroscedasticity is not present using non-constant variance score test. Also, the

variance inflation factor on each variable of the full model is smaller than 2 except for GDP per capita (2.46) and Internet diffusion (2.51) that are strongly correlated with each other but neither contributes significantly to model predictions.

In short, as shown in Table 2 and 3 in the main text, cultural openness is more closely associated with cultural values than the non-cultural factors.
