# Supplementary material for: Cultural values and cross-cultural video consumption on YouTube
Source: PLoS One. 2017 May 22;12(5):e0177865. doi: 10.1371/journal.pone.0177865 (PMC5439684; doi:10.1371/journal.pone.0177865)
Supplement: S2 Text — (PDF) [file pone.0177865.s003.pdf]

OLS regression analyses including a country's degree in the international migration network as a control variable.

As mentioned in the main text, we check the robustness of our results by adding degrees in the migration network as a control variable:

Table 1. OLS regression model of cultural betweenness among 58 countries.

	Full model	Non-culture Model	Culture Model
**Intercept**	-0.455*	-0.005	-0.339*
	(0.171)	(0.073)	(0.147)
**Non-cultural factors**			
GDP per capita (log-transformed)	0.013	0.003	
	(0.223)	(0.233)	
Language eigenvector centrality	0.089	0.147	
	(0.084)	(0.078)	
Number of Internet users	0.091	0.013	
	(0.240)	(0.241)	
Degree in Migration Network	0.193	0.184	
	(0.119)	(0.101)	
**Cultural values**			
Individualism (IDV)	0.240		0.426**
	(0.167)		(0.123)
Uncertainty avoidance (UAI)	-0.068		-0.061
	(0.123)		(0.113)
Power distance (PDI)	0.439**		0.373*
	(0.155)		(0.147)
Masculinity (MAS)	0.222		0.250*
	(0.134)		(0.124)
**Sample size (number of countries)**	58	58	58
**Model-fit indices**			
R^2	0.417	0.164	0.396
Adjusted R^2	0.336	0.099	0.350

Note: * $p < .05$, ** $p < .01$, *** $p < .001$. Unstandardized coefficients are reported with standard errors in parentheses. In order to compare coefficients, variables included in the analyses were rescaled to the unit interval, meaning that the minimum value is 0 and the maximum value is 1.

Table 2. OLS regression model of cultural closeness among 58 countries.

	Full model	Non-culture Model	Culture Model
**Intercept**	0.106	0.353***	0.285**
	(0.102)	(0.051)	(0.100)
**Non-cultural factors**			
GDP per capita (log-transformed)	0.122	0.116	
	(0.133)	(0.163)	
Language eigenvector centrality	0.072	0.170**	
	(0.050)	(0.055)	
Number of Internet users	0.082	-0.004	
	(0.143)	(0.168)	
Degree in Migration Network	0.209**	0.152*	
	(0.071)	(0.071)	
**Cultural values**			
Individualism (IDV)	0.104		0.328***
	(0.100)		(0.084)
Uncertainty avoidance (UAI)	-0.269***		-0.237**
	(0.074)		(0.077)
Power distance (PDI)	0.344***		0.236*
	(0.093)		(0.100)
Masculinity (MAS)	0.254**		0.270**
	(0.080)		(0.084)
**Sample size (number of countries)**	58	58	58
**Model-fit indices**			
R^2	0.618	0.342	0.452
Adjusted R^2	0.555	0.291	0.411

Note: * $p < .05$, ** $p < .01$, *** $p < .001$. Unstandardized coefficients are reported with standard errors in parentheses. In order to compare coefficients, variables included in the analyses were rescaled to the unit interval, meaning that the minimum value is 0 and the maximum value is 1.

Table 3. OLS regression model of cultural openness (the composite measure) among 58 countries.

	Full model	Non-culture Model	Culture Model
**Intercept**	0.282	0.488	0.363*
	(0.160)	(0.077)	(0.139)
**Non-cultural factors**			
GDP per capita (log-transformed)	-0.201	-0.262	
	(0.208)	(0.247)	
Language eigenvector centrality	0.002	0.163	
	(0.079)	(0.083)	
Number of Internet users	0.305	0.312	
	(0.225)	(0.255)	
Degree in Migration Network	0.115	0.089	
	(0.125)	(0.107)	
**Cultural values**			
Individualism (IDV)	0.269		0.403**
	(0.156)		(0.117)
Uncertainty avoidance (UAI)	-0.466***		-0.439***
	(0.115)		(0.104)
Power distance (PDI)	0.485**		0.464**
	(0.145)		(0.139)
Masculinity (MAS)	0.230		0.186
	(0.125)		(0.115)
**Sample size (number of countries)**	58	58	58
**Model-fit indices**			
R^2	0.454	0.120	0.423
Adjusted R^2	0.363	0.052	0.378

Note: * $p < .05$, ** $p < .01$, *** $p < .001$. Unstandardized coefficients are reported with standard errors in parentheses. In order to compare coefficients, variables included in the analyses were rescaled to the unit interval, meaning that the minimum value is 0 and the maximum value is 1.
